# Magnetic Fluctuations Entrain the Circadian Rhythm of Locomotor Activity in Zebrafish: Can Cryptochrome Be Involved?

**DOI:** 10.3390/biology11040591

**Published:** 2022-04-13

**Authors:** Viacheslav V. Krylov, Evgeny I. Izvekov, Vera V. Pavlova, Natalia A. Pankova, Elena A. Osipova

**Affiliations:** 1 Papanin Institute for Biology of Inland Waters, Russian Academy of Sciences, 152742 Borok, Russia; eiizvekov@ibiw.ru (E.I.I.); vera@ibiw.ru (V.V.P.); stellaria1985@yandex.ru (N.A.P.); osipova@ibiw.yaroslavl.ru (E.A.O.); 2 Scientific and Technological Center of Unique Instrumentation, Russian Academy of Sciences, 117342 Moscow, Russia

**Keywords:** *Danio rerio*, magnetic field, circadian rhythm, swimming speed, cryptochrome

## Abstract

**Simple Summary:**

Most physiological processes are subject to biological circadian rhythms maintained by a complex cascade of biochemical events. The circadian rhythmicity of behavior allows organisms to use energy and resources optimally under changing environmental conditions. To that end, endogenous circadian rhythms are synchronized with external pacemakers (zeitgebers), especially daily changes in illumination. In the 1960s, it was assumed that, in addition to this primary photic cue, animals can use diurnal geomagnetic variation as a secondary zeitgeber. Earlier research found that slow magnetic fluctuations can affect some behavioral endpoints of circadian rhythms by modulating an organism’s physiological state. However, no direct experiments to test such an entrainment of biological clocks by artificial magnetic fields were performed due to the technical difficulty of eliminating natural geomagnetic variation. For the first time, we carried out such tests in a fully controlled magnetic environment using zebrafish as a research model. The experimental treatments included various light/dark cycles and continuous illumination coupled with pre-recorded natural geomagnetic variations. The obtained results indicate that slow magnetic fluctuations can entrain endogenous rhythmical activity in vertebrates. Probably, cryptochromes play a key role in this process. This research provides promising opportunities for the magnetic control of circadian processes, e.g., correcting circadian dysfunctions.

**Abstract:**

In the 1960s, it was hypothesized that slow magnetic fluctuations could be a secondary zeitgeber for biological circadian rhythms. However, no comprehensive experimental research has been carried out to test the entrainment of free-running circadian rhythms by this zeitgeber. We studied the circadian patterns of the locomotor activity of zebrafish (*Danio rerio*) under different combinations of light regimes and slow magnetic fluctuations, based on a record of natural geomagnetic variation. A rapid synchronization of activity rhythms to an unusual 24:12 light/dark cycle was found under magnetic fluctuations with a period of 36 h. Under constant illumination, significant locomotor activity rhythms with 26.17 h and 33.07 h periods were registered in zebrafish exposed to magnetic fluctuations of 26.8 h and 33.76 h, respectively. The results reveal the potential of magnetic fluctuations for entrainment of circadian rhythms in zebrafish and genuine prospects to manipulate circadian oscillators via magnetic fields. The putative mechanisms responsible for the entrainment are discussed, including the possible role of cryptochromes.

## 1. Introduction

The majority of processes in living organisms follow a 24 h cycle. This circadian rhythm fits an organism’s effective use of energy and resources in an ever-changing environment [1]. A complex of biochemical reactions known as a transcription–translation feedback loop is the core endogenous oscillator which provides a rhythm close to 24 h [2]. In most vertebrates, this is a negative feedback loop, with the transcription factors CLOCK and BMAL operating to modulate expression of period (*Per*) and cryptochrome (*Cry*) genes. These, in turn, repress transcription to allow the start of a new cycle of the feedback loop [1,2]. The described endogenous rhythms of intracellular circadian oscillators are transferred to higher levels of organization and allow an organism to adjust its processes to the anticipated conditions for a given time of day [3].

A natural daily light/dark cycle is the primary external circadian pacemaker (i.e., zeitgeber) coupling endogenous circadian rhythms with the environment [4]. Other external periodic influences can also act as zeitgebers under constant light or darkness, such as diurnal temperature oscillations [5,6,7]. It is known that, in zebrafish, scheduled feeding can also serve as a zeitgeber [8,9,10] due to a link between the endogenous circadian clock and associative memory [11]. These influences entrain free-running rhythms—phase shifts or changes in the period of biological rhythms corresponding to changes in periodic external influences. In contrast, many other external stimuli can affect the amplitude of circadian rhythms. Such influences modulate an organism’s physiological state, which diminishes or enhances the endpoints of circadian rhythms [12,13].

Since 1960, magnetic fields have been of special interest among the environmental factors capable of affecting circadian rhythms [14,15,16,17]. Recent publications on this issue report that static and weak radiofrequency magnetic fields lead to changes in the period of the circadian rhythm of locomotor activity in cockroaches [18] and *Drosophila* [19]. An increased acrophase level and a phase shift in locomotor activity rhythms were also revealed in rats after exposure to radiofrequency magnetic fields [20]. In addition to behavioral rhythms, magnetic fields altered the expression of circadian clock genes [21,22]. The exposure of zebrafish fibroblasts to magnetic fields has been shown to cause an increase in *Cry1aa* expression and the shifting of the *Cry1aa* and *Per1b* oscillation phases [23]. Similarly, the exposure of zebrafish larvae to the same magnetic fields between 11 and 14 days post-fertilization led to the desynchronization of the *Cry1aa* and *Per1b* circadian oscillations [23].

Brown suggested that slow magnetic fluctuations could be a secondary zeitgeber for biological circadian rhythms [14]. This suggestion emerged from the fact that there is natural diurnal geomagnetic variation; the strength of Earth’s geomagnetic field changes daily from approximately a few tens of nanotesla (nT) at mid-latitudes to values of approximately 200 nT near the magnetic equator within a 24 h period [24]. Recent findings, that the biological effects of geomagnetic disturbances depend on synchronization with the peak of diurnal geomagnetic variation [25,26], support this suggestion.

In recent decades, more clarity has emerged regarding possible pathways that might be responsible for the magnetic sensitivity of the circadian system in an organism. The cryptochromes, which are key elements of organisms’ transcription–translation negative feedback loop, are considered the most probable biological magnetodetector [27]. It is suggested that the inclination of the external magnetic field can affect the electron spins in cryptochrome’s radical pairs, thereby changing the functional state of the molecule [28]. In this regard, cryptochrome-1 and cryptochrome-2 are regarded as temperature-insensitive magnetodetectors [29] that may provide temperature compensation for the circadian clock [30]. Magnetoreceptor protein can be another molecule involved in protein–protein interactions for the transduction of magnetic stimuli from cryptochromes to higher levels [31,32]. The inhibition of its expression has been shown to result in the disruption of circadian behavior in fruit flies [33].

Despite the increasing number of publications on this subject in recent years, all previous studies evaluated only changes in the endpoints of circadian rhythms. However, no comprehensive experiments have yet been performed to evaluate the entrainment of free-running circadian rhythms by slow magnetic fields. Such research might clarify whether slow magnetic fluctuations can only affect the physiological state and mask or enhance circadian rhythms, or whether they are able to entrain the endogenous circadian oscillators, which opens prospects for manipulating circadian clocks via magnetic fields. The present paper reports the first results in this field.

We used wild-type zebrafish (*Danio rerio*) as a model organism. The pineal gland in the brain of this species drives the rhythmic production of melatonin [34]. However, the circadian oscillators in different zebrafish tissues can keep unrelated rhythms entrained directly by an external light–dark zeitgeber [35,36,37]. The lack of a centralized pacemaker subjugating all other oscillators in zebrafish could increase the chance to detect changes in circadian rhythms caused by a magnetic influence. We analyzed the locomotor activity, which is known as a precise indicator of circadian rhythmicity in this species [11], upon exposure to magnetic fields.

## 2. Materials and Methods

All experimental protocols have been approved by the Institutional Animal Care and Use Committee at the Papanin Institute for Biology of Inland Waters.

### 2.1. Zebrafish Maintenance

Wild-type zebrafish (AB strain) were obtained from a commercial distributer and maintained in the Laboratory of Physiology and Toxicology (Papanin Institute for Biology of Inland Waters, Russian Academy of Sciences). Before experimentation, *D. rerio* were kept together for two months in 70 L aquaria at 24 °C. A 16:8 h light/dark cycle was used, as it was more preferable for zebrafish cultivation [38]. *D. rerio* were fed daily at different times between 12:00 and 16:30. Males and females at the age of approximately four months (2.99 ± 0.17 cm, 0.26 ± 0.02 g, *n* = 24) were used for experimentation. Each zebrafish was used only for a single replication.

### 2.2. Timed Backlight

In order to provide backlit illumination for the experiments, a lightbox was constructed from a series of LEDs, aluminum plates, and matte plexiglass. LED plates were created by adhering 32 LEDs to an aluminum plate so that each aquarium would be backlit by four infrared LEDs (3 W, 940 nm) and four white-color LEDs (3 W, 4500 K). Each LED plate was mounted 10 cm under a lightbox cover made of matte plexiglass that diffused the light. Lighting modes were controlled via time relays (DH-48S-S, Omron, Kyoto, Japan) which used KMI-10910 (IEK, Moscow, Russia) contactors to supply power, by Qh-60LP18 power suppliers (Shenzhen Chanzon Technology, Shenzhen, China), to the LEDs. Constant illumination and 16:8 and 24:12 light/dark cycles were used in the experiments.

### 2.3. Magnetic Fluctuations

The following magnetic fluctuations were used for the experiments:

1. Natural diurnal geomagnetic variation. This is represented by magnetic fluctuations of about 30 nT within a 24 h period. This variation was recorded in the X-, Y-, and Z-directions throughout the experiment using an NV0302A magnetometer (ENT, St Petersburg, Russia);
2. Experimental magnetic fluctuations simulating increased diurnal geomagnetic variation within 26.8 h, 33.76 h, and 36 h periods. We used a sample record of diurnal geomagnetic variation in the X-, Y-, and Z-directions that were made close to the laboratory to generate these magnetic oscillations. The sample record intensity was enhanced to about 100–150 nT for each X-, Y-, and Z-direction. This exposure allowed for more pronounced periodic changes in the magnetic background without exceeding the level of natural geomagnetic storms. The period of the magnetic variation for different experimental groups was also increased to 26.8 h, 33.76 h, or 36 h by a signal prolongation.

A setup described in detail by Krylov et al. [39] was used to generate experimental magnetic fluctuations. It was assembled on a PC workstation and consisted of the following items:

(1) A three-component fluxgate magnetometer NV0302A (ENT, St Petersburg, Russia) providing analogous signals proportional to the strength of the geomagnetic field and its variations;
(2) An LTR11 analog-to-digital and an LTR34-4 digital-to-analog signal converter (L-card, Moscow, Russia);
(3) A coil system consisting of three pairs of mutually orthogonal Helmholtz coils (0.5 m in diameter, 700 turns of 0.2 mm copper wire in each coil) made by the Schmidt Institute of Physics of the Earth (www.ifz.ru) (accessed on 10 April 2022).

The direction of each Helmholtz coil pair was the same as the direction of the geomagnetic field components. Natural geomagnetic disturbances and diurnal geomagnetic variations were compensated within the Helmholtz coil systems in the frequency range of up to 5 Hz, based on an NV0302A magnetometer signal. The compensation of magnetic fluctuations is shown in the Supplementary Video S1. The industrial alternating magnetic fields of 50 Hz were less than 10 nT and did not appear in the harmonics. The generated signals in the Helmholtz coil system’s working volume were checked using a control magnetometer NV0599C (ENT).

### 2.4. Experimental Conditions and Procedure

All experimentation was conducted in a remote laboratory building free of working staff in order to eliminate possible circadian rhythm disturbance caused by daily human activities. Each fish was placed in a single custom glass aquaria (15 × 20 cm, height 23 cm) filled with 10 cm of water. Water temperature during the experiments was 21 °C, as adult zebrafish show the most robust rhythm of locomotor activity at the temperatures of 20–21 °C [6,40]. Four aquaria were installed above a lightbox. Screens made of opaque white plastic were placed between the adjacent aquaria so that fish could not see conspecifics. The lightbox with the aquaria was located in a system of Helmholtz coils.

**Figure 1:**
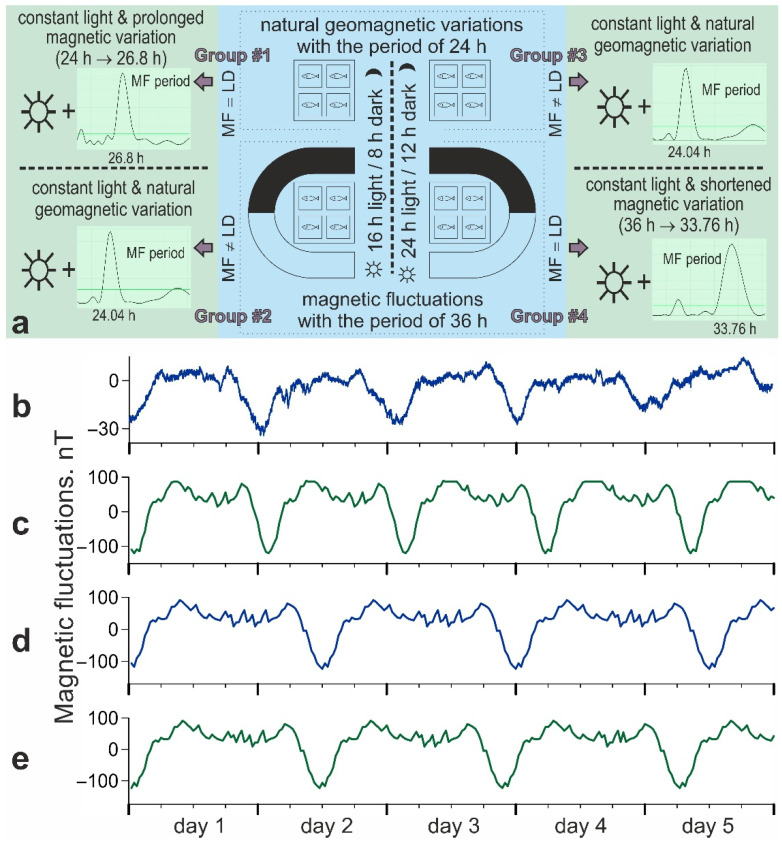
Scheme of the experiments (**a**) and magnetic fluctuations applied to zebrafish (**b**–**e**): natural diurnal geomagnetic variation, averaged from several 5-day periods in time-separated replications (**b**); magnetic variation with 26.8 h period used at the second stage of experiments, extended relative to the initial 24 h period (**c**); magnetic variation with 36 h period used at the first stage of experiments (**d**); magnetic variation with 33.76 h period used at the second stage of experiments, shortened relative to the initial 36 h period (**e**).

The experiments were carried out in two stages (Figure 1). At the first stage, four groups of fish were kept for 4.5 days under the following conditions:

Group #1. 16:8 light/dark cycle and natural diurnal geomagnetic variation (24 h period). The periods of light and magnetic stimuli coincide and correspond to the natural ones;
Group #2. 16:8 light/dark cycle and artificially prolonged magnetic variation (36 h period). The light and magnetic stimuli periods do not coincide; the main zeitgeber period corresponds to the natural one;
Group #3. 24:12 light/dark cycle and natural diurnal geomagnetic variation (24 h period). The light and magnetic stimuli periods do not coincide; the main zeitgeber period does not correspond to the natural one;
Group #4. 24:12 light/dark cycle and artificially prolonged magnetic variation (36 h period). The periods of light and magnetic stimuli coincide and do not correspond to the natural ones.

All groups were kept under constant illumination for 4.5 days at the second stage to detect free-running activity rhythms. Zebrafish from groups #2 and #3 (unequal periods of light and magnetic stimuli at the first stage of the experiment) were then exposed to a natural diurnal geomagnetic variation with a period of 24 h. Meanwhile, the individuals from group #1 (24 h period of light and magnetic stimuli at the first stage) were exposed to extended magnetic variation with a 26.8 h period. Zebrafish from group #4 (the 36 h period of light and magnetic stimuli at the first stage) were later subjected to magnetic variation with a shorter 33.76 h period.

The water was constantly renewed via two 4 mm openings in the wall of each aquarium at 3 and 10 cm height from the bottom. Water flowed by gravity from a 200 L plastic barrel placed upstairs and through silicone hoses connected to the bottom openings of the aquaria. Water aeration and temperature control for all aquaria were carried out in the barrel. Excess water was drained to the sewer through the top opening to ensure a constant level of 10 cm. Water from different aquaria was never mixed or reused.

At the beginning of the experiment, a 1 cm^3^ piece of slow-release gel food block “Tetra Holiday” (Tetra GmbH, Melle, Germany) was placed on the bottom of each aquarium to prevent the influence of the feeding schedule on circadian behavior; thereby, the zebrafish had free access to food during the whole study.

Fish movements in the horizontal plane were registered with IP cameras (TR-D1140, Trassir, Shenzhen, China) equipped with IR-corrected varifocal lenses (TR-L4M2.7D2.7-13.5IR, Trassir, Shenzhen, China) and mounted above the aquaria. Night and day videos were recorded in black and white at 25 frames per second with a 2592 × 1520-pixel resolution. The video signals were transmitted through a switch (T1500-28PCT, TP-Link, Shenzhen, China) to a video recorder server (MiniNVR AF16, Trassir, Shenzhen, China).

The experiment was performed in 3 replications between 31 July 2020 and 17 September 2020. Thereby, 216 hours of video records from 12 zebrafish were then processed.

### 2.5. Data Processing

An approach proposed by Audira et al. [12] was used for data processing. One-minute video clips were cut from the primary video record for every half-hour (from the 15th to the 16th and the 45th to the 46th minute of each hour). Such a duration has proved to be sufficient for the statistical analysis of locomotor activity, with the data appropriately describing circadian rhythms. The open-source software idTracker [41] was used to process each one-minute video file. The software provided X and Y coordinates reflecting the center of the fish body for each frame. Before the processing, the trajectory data were filtered using the “minimal distance moved” method to eliminate slight “apparent” movements of the fish [42]. The minimal distance threshold was set at 2.6 mm. Then, based on this information, the average swimming speed was calculated.

All data had a normal distribution (Shapiro–Wilk W-test, *p* > 0.05). Differences between the values during the light and dark phases were evaluated with a paired t-test, i.e., the average value for each fish in the light phase was compared with the values obtained from the same individuals in the dark phase (*n* = 12). The time series were analyzed with RhythmicAlly software [43]. The linear trend was subtracted from the time series, and the data were smoothed with a moving average window of 7 samples before analysis. Periods in zebrafish locomotor activity were analyzed using the Lomb–Scargle periodogram [44].

## 3. Results

At the first stage of the experiments, almost all zebrafish groups displayed significantly higher swimming activity during the light phase compared to the dark phase. The only exception was group #3, which was kept under a prolonged 24:12 light/dark cycle coupled with natural diurnal geomagnetic variation, which showed no significant difference between the dark and light phases (Table 1).

**Figure 2:**
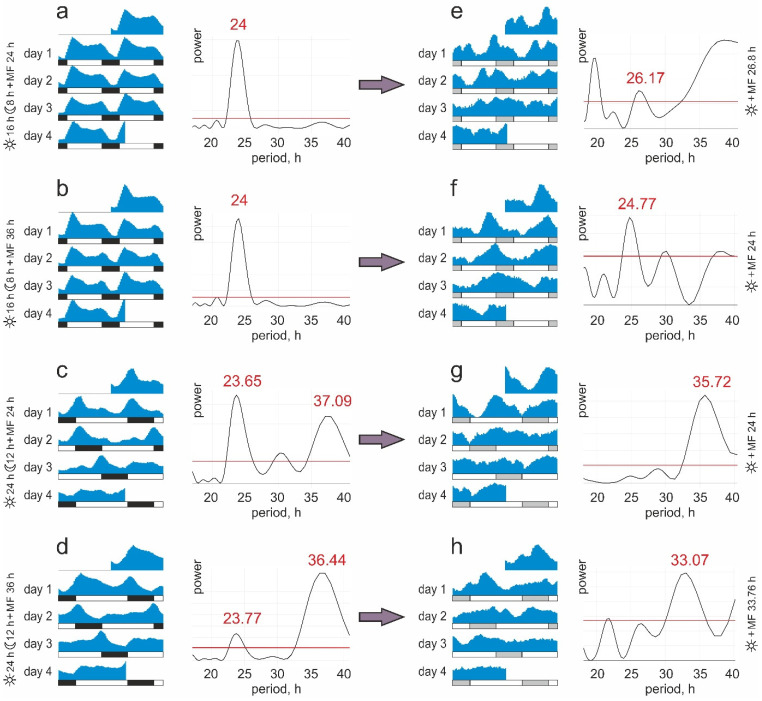
Representative double-plotted actograms and Lomb–Scargle periodograms of gross locomotor activity of zebrafish maintained at different combinations of light regime and magnetic fluctuations (MF). Group #1: (**a**) 16:8 light/dark cycle and 24 h MF at the first stage of experiments with subsequent transfer (**e**) to 26.8 h MF under constant illumination. Group #2: (**b**) 16:8 light/dark cycle and 36 h MF at the first stage with subsequent transfer (**f**) to 24 h MF under constant illumination. Group #3: (**c**) 24:12 light/dark cycle and 24 h MF at the first stage with subsequent transfer (**g**) to 24 h magnetic fluctuations under constant illumination. Group #4: (**d**) 24:12 light/dark cycle and 36 h MF at the first stage with subsequent transfer (**h**) to 33.76 h MF under constant illumination. The bars below the actograms represent the light/dark cycle; open and black bars represent the light and dark phases of the cycle, respectively; open and gray bars represent the light and dark phases of the cycle applied before constant illumination, respectively. Significant peaks (*p* < 0.05) exceed the red horizontal line on the periodograms.

In groups of zebrafish exposed to the 16:8 light/dark cycle, only 24 h period locomotor activity was manifested (periodogram analysis in Figure 2a,b). It is noteworthy that magnetic fluctuations with a 36 h period did not affect this rhythm (Figure 2b), which evidences the primary role of the photic cue for both of these groups.

For groups #3 and #4, the usual light/dark cycle was suddenly extended by one-and-a-half times at the start of experimentation (Figure 2c,d), which caused dynamic processes of circadian rhythm adaptation in zebrafish. Endogenous oscillators still dictated the usual 24 h rhythmicity, but it no longer corresponded to the new realities of the 24:12 light/dark cycle. Accordingly, two significant periodic components close to 24 and 36 h were revealed in the locomotor activity of these groups of zebrafish (Figure 2c,d). The period of 23.65 h prevailed in zebrafish exposed to conflicting light and magnetic zeitgebers (Figure 2c). For this reason, the increased locomotor activity was registered during the dark phase, resulting in a lack of significant differences between the light and dark phases in this group (Table 1). However, by the fourth day, zebrafish tuned their diurnal swimming pattern to a new rhythm; the actogram clearly shows the expected decline of activity in the dark phase, and its rise when the lights are on, which is also manifested as a significant secondary peak (37.09 h) in the periodogram (Figure 2c). Such a long-period locomotor activity with a 36.44 h period was most pronounced in zebrafish exposed to the 24:12 light/dark cycle coupled with 36 h magnetic fluctuations (Figure 2d). It is noteworthy that a relatively quick adjustment of the zebrafish to a new rhythm occurred when exposed simultaneously to the action of a long-period light zeitgeber and extended magnetic variation. In this case, a robust significant rhythm with a period of 36.44 h was established on the actograms since the second day. Thus, magnetic fluctuations with the same period helped zebrafish quickly tune their circadian system to a new light/dark cycle.

At the second stage of the experiment performed under constant illumination, all groups of zebrafish showed clear signs of free-running rhythmical activity. The entrainment of this rhythm by magnetic fields was expected to manifest in groups #1 and #4, where magnetic fluctuations with a new period different from the initial were applied. Indeed, the zebrafish from group #1, which were previously maintained under a 16:8 light/dark cycle with natural geomagnetic variation, now revealed a significant peak at 26.17 h, close to a new magnetic zeitgeber period of 26.8 h (Figure 2e). The entraining capacity of magnetic stimuli was also clearly seen in zebrafish from group #4, previously maintained under a 24:12 light/dark cycle accompanied by magnetic fluctuations with a period of 36 h. In this group, a reduction in the period of magnetic zeitgeber to 33.76 h was matched by a corresponding reduction in the period of locomotor activity, to a value of 33.07 h (Figure 2h). Meanwhile, in zebrafish previously kept under a 16:8 light/dark cycle with 36 h magnetic fluctuations, the period of free-running activity at the second stage of the experiments (24.77 h) remained close to the natural diurnal geomagnetic variation (Figure 2f). Notably, in group #3, featured by a significant mismatch between the natural geomagnetic variation (24 h) and the endogenous rhythm (36 h), the zebrafish still maintained a long-period activity rhythm of 35.72 h (Figure 2g). This is not surprising, considering that even light stimuli could not immediately readjust the endogenous rhythm, as seen in Figure 2c,d. It appears that slow magnetic fluctuations successfully entrained the circadian rhythmicity of zebrafish locomotor activity only when their periods did not differ significantly from the endogenous rhythm (Figure 2e,h).

## 4. Discussion

The study findings provide experimental evidence that slow magnetic fluctuations can entrain the free-running activity rhythms in *D. rerio*. The locomotor activity of zebrafish with a period of 26.17 h in the group exposed to magnetic fluctuations with a 26.8 h period (Figure 2a) is remarkable, as we found no mention of the locomotor activity rhythms longer than 25 h in zebrafish maintained under constant illumination without additional zeitgebers. It was reported that such free-running periods vary from 23.5 to 24.5 h, depending on the water temperature [40]. Another study revealed the shortening of daily rhythms in zebrafish locomotor activity to 22.9–23.6 h under constant dim light [6]. The free-running rhythm of locomotor activity in zebrafish also became shorter (22.9 ± 0.5 h) under ultradian 45:45 min light/dark cycles [45].

The obtained results characterize the diurnal geomagnetic variation as a secondary environmental zeitgeber which is synchronized with the dominant light/dark zeitgeber in most natural environments. However, the geomagnetic zeitgeber might become the only “environmental clock” in thermostable and aphotic places such as caves, deep-sea trenches, etc.

Magnetic stimuli likely influence endogenous circadian oscillators via cryptochromes. It is suggested that the light-induced redox transition of electrons between flavin adenine dinucleotide and tryptophan residues forms long-lived radical pairs in cryptochromes [46,47]. Following this suggestion, relatively weak magnetic fields may affect the spins of unpaired electrons in these radical pairs, resulting in a change in the singlet and triplet yields of radical-pair reactions in cryptochromes [28]. The hypothesis of light-dependent magnetoreception in birds [27] and other animals [48] is based on this mechanism. The crucial role of cryptochromes in the transcription–translation feedback loop makes them the main candidates for a magneto-sensitive element responsible for the influence of magnetic fields on circadian rhythms.

Due to duplication events that occurred during teleost evolution [49], the zebrafish genome contains several homologs of cryptochrome genes [32], among which *Cry1a* acts as components of the core clock mechanism [50]. The study of possible *Cry1b* involvement in the magnetic regulation of circadian rhythms seems promising, as it is a homolog of *Cry1a* but is not induced by light [51]. *Cry4* is also of interest as the most probable biological detector of magnetic fields [52]. The above mechanisms could be responsible for the entrainment of circadian rhythms by slow magnetic fluctuations in zebrafish. Overall, the possibility of such entrainment opens up prospects for the effective control of circadian processes using magnetic stimuli, and provides a deeper insight into the generation and maintenance of biological rhythms. In addition, the use of slow magnetic variations can be helpful for correcting circadian rhythm disorders, which suggests the need for further research in this area. Some recommendations for the generation of slow magnetic fluctuations are given in the Supporting Information (File S1).

## 5. Conclusions

Over the past decades, the diurnal geomagnetic variation displaying pronounced circadian rhythmicity has been a focus of attention as a putative non-photic zeitgeber. Our studies with pre-recorded geomagnetic variations indicate that slow magnetic fluctuations can entrain endogenous locomotor activity in zebrafish even under continuous illumination. Magnetic stimuli also dramatically facilitate the entrainment of this rhythmical activity to an unusual long-period light/dark cycle. The emerging opportunities to influence the circadian clock via magnetic fluctuations call for further experimental studies in the field, including those aimed at clarifying the possible role of cryptochromes.

## Figures and Tables

**Table 1:** Studied behavioral endpoints in zebrafish at the light and dark phases during the first stage of experiments.

Experimental Group	Average Swimming Speed (cm/s)	Meandering (°/cm)	Average Angular Velocity (°/s)	Freezing Time (%)	Swimming Time (%)	Rapid Movement Time (%)
#1 16-8 L/D MF period 24 h	2.33 ± 0.24 0.96 ± 0.10 *	35.31 ± 1.77 69.77 ± 4.99 *	78.60 ± 9.77 47.56 ± 4.19 *	26.41 ± 4.33 71.46 ± 2.45 *	72.44 ± 4.06 28.29 ± 2.40 *	1.15 ± 0.43 0.25 ± 0.07
#2 16-8 L/D MF period 36 h	2.34 ± 0.23 1.05 ± 0.08 *	39.04 ± 3.74 60.50 ± 5.82 *	70.80 ± 5.25 47.48 ± 2.49 *	33.88 ± 5.74 67.69 ± 2.81 *	64.11 ± 5.66 32.05 ± 2.78 *	2.01 ± 0.41 0.26 ± 0.04 *
#3 24-12 L/D MF period 24 h	1.93 ± 0.18 1.97 ± 0.14	59.27 ± 12.10 45.35 ± 2.79	77.22 ± 7.40 86.41 ± 8.24	43.44 ± 5.52 40.87 ± 3.25	55.16 ± 5.56 58.39 ± 3.19	1.38 ± 0.22 0.74 ± 0.19
#4 24-12 L/D MF period 36 h	2.19 ± 0.19 1.54 ± 0.10 *	42.92 ± 3.22 52.84 ± 8.09	70.15 ± 5.27 63.36 ± 4.53	33.04 ± 5.15 52.70 ± 3.01 *	65.71 ± 4.98 46.55 ± 3.05 *	1.25 ± 0.23 0.66 ± 0.16

Note: Data are given as means ± standard error. Over the dash—value at the light phase; under the dash—value at the dark phase. L = light; D = dark; MF = magnetic fluctuation. * Significant differences between values at the light and dark phases (a paired *t*-test, *p* < 0.05, *n* = 12).

## Data Availability

All data generated or analyzed during this study are included in this published article (Data S1).

## Supplementary Materials

The following supporting information can be downloaded at: https://www.mdpi.com/article/10.3390/biology11040591/s1, Data S1: time series of zebrafish activity; Vedio S1: the generation and compensation of magnetic fluctuations; File S1: Supporting information. References [53,54] are cited in the Supplementary Materials.
