# Supplementary material for: Magnetic Fluctuations Entrain the Circadian Rhythm of Locomotor Activity in Zebrafish: Can Cryptochrome Be Involved?
Source: Biology (Basel). 2022 Apr 13;11(4):591. doi: 10.3390/biology11040591 (PMC9025847; doi:10.3390/biology11040591)
Supplement: Supplementary file 1 [file biology-11-00591-s001.zip › File S1.pdf]

Supporting information

Recommendations for generation of slow magnetic fluctuations

The study revealed several conditions to be met when reproducing the pre-recorded natural or artificially generated slow magnetic fluctuations to entrain the endogenous circadian oscillators. First, it is necessary to avoid a superposition of the reproduced fluctuations with natural geomagnetic variations, which might affect the biological response (Krylov, 2021).

1. The natural geomagnetic variation can be eliminated through its active compensation in the system of Helmholtz coils, as applied in this work. Alternatively, shields made of soft magnetic materials can be used. In the latter case, in addition to a zeitgeber magnetic signal, it is necessary to provide a static magnetic field corresponding to the intensity and direction of the local geomagnetic field to avoid the possible effects of hypomagnetic conditions (Binhi and Prato, 2017).

Since the active compensation using feedback from a reference magnetometer can be challenging to implement, passive compensation may also be used. Though the pattern of diurnal geomagnetic variation is changeable, its general predictability allows for an added signal to account for these daily changes. The intensity and timing of this compensatory signal should follow the expected pattern of diurnal geomagnetic variation, while its direction must be opposite for each orthogonal magnetic component. Then the diurnal variation will be successfully compensated by fluctuations inherent in the experimental signal. However, it should be stressed that this method does not work well during geomagnetic storms or sudden changes in the pattern of diurnal variation. These events can notably modulate the resultant magnetic field affecting the test organisms. Therefore, active compensation is preferable to passive.

2. Three-component records of the diurnal geomagnetic variation made near the test site during magnetically quiet times can be used as the basis for signal reproduction. This case should account for the dynamics of natural magnetic variations. There should not be a significant phase shift between the new fluctuation generated and the experienced before diurnal geomagnetic variation at the start of experimental treatment.

3. Sometimes (e.g., in the case of passive compensation), it seems advisable to amplify the reproduced signal to increase magnetic variation potency, thus separating it from possible background noise. The limit of natural magnetic disturbances (about 300 nT) should not be exceeded as a physiological sensitivity window may exist (Wiltschko, 1978). If the original signal contains sudden amplitude jumps, after amplification, they could become comparable to the natural geomagnetic events, and the test organisms might perceive them as environmental time cues. In this case, the experimental signal will need to be smoothed before amplification, retaining only the target slow magnetic fluctuations.

4. The dynamics of magnetic fields during the experiments should be monitored with a magnetometer located close to the test site. It is necessary to control both the generated signals and possible temporary disturbances of the local magnetic background.

REFERENCES

Binhi, V.N. & Prato, F.S. Biological effects of the hypomagnetic field: An analytical review of experiments and theories. *Plos ONE* **12**(6): e0179340 (2017).

Krylov, V.V. Influence of geomagnetic disturbances at different times of day on locomotor activity in zebrafish (*Danio rerio*). *Clocks & Sleep* **3,** 624–632 (2021).

Wiltschko, W. Further analysis of the magnetic compass of migratory birds. *Animal migration, navigation and homing.* Berlin: Springer (1978).
